# Treatment Patterns and Clinical Outcomes in Children with *Stenotrophomonas maltophilia* Infections

**DOI:** 10.3390/microorganisms14092087

**Published:** 2026-09-18

**Authors:** Carly Mitchell, Tania Thomas, Angelica Rivera-Agosto, Gustavo R. Alvira-Arill, Ashlan J. Kunz Coyne, Stephen A. Thacker, Krutika Mediwala Hornback, Taylor Morrisette

**Affiliations:** 1 Department of Clinical Pharmacy & Outcomes Sciences, Medical University of South Carolina College of Pharmacy, Charleston, SC 29425, USA; carlym3129@gmail.com (C.M.); thomatan@musc.edu (T.T.); riveraag@musc.edu (A.R.-A.); alviraar@musc.edu (G.R.A.-A.); 2 Department of Pharmacy Services, Medical University of South Carolina Health, Charleston, SC 29425, USA; mediwala@musc.edu; 3 Department of Pharmacy Practice and Science, University of Kentucky College of Pharmacy, Lexington, KY 40536, USA; ashlan.kunz.coyne@uky.edu; 4 Department of Pediatrics, Division of Infectious Diseases, Medical University of South Carolina Health, Charleston, SC 29425, USA; thackest@musc.edu

**Keywords:** *Stenotrophomonas maltophilia*, pediatrics, resistant Gram-negative bacteria

## Abstract

*Stenotrophomonas maltophilia* is an opportunistic, multidrug-resistant pathogen commonly associated with respiratory and bloodstream infections. Owing to extensive intrinsic and acquired antimicrobial resistance and limited pediatric-specific evidence, contemporary management relies heavily on extrapolation from adult data, underscoring the need to better characterize real-world treatment practices and outcomes in children. This was a retrospective, observational cohort that included hospitalized patients < 18 years of age with a positive *S. maltophilia* culture who received active antimicrobial therapy, excluding cases determined by the treating provider to represent colonization. Outcomes assessed included in-hospital mortality, adverse drug effects, and clinical success rates using descriptive statistics. Forty pediatric patients (median [IQR] age of 2.7 [0.7–7.9] years) were included. Respiratory infections accounted for 77.5% of cases, and sulfamethoxazole-trimethoprim monotherapy was the most common treatment regimen (70.0%). Five patients (12.5%) experienced in-hospital mortality, three patients (7.5%) experienced adverse effects related to treatment, and all patients exhibited clinical success. Larger, multicenter observational and prospective studies are warranted to strengthen evidence-based therapeutic approaches for pediatric patients with *S. maltophilia* infections.

## 1. Introduction

*Stenotrophomonas maltophilia* is a non-fermenting, Gram-negative bacillus that inhabits a wide range of environmental settings associated with a diverse range of infections [1,2]. The organism most commonly causes respiratory and bloodstream infections and is frequently identified as an opportunistic pathogen among hospitalized or immunocompromised patients [2,3]. Reported risk factors for *S. maltophilia* infections include underlying pulmonary conditions, indwelling medical devices, immunosuppression, prolonged hospitalization, and prior exposure to broad-spectrum antibiotics such as carbapenems [4,5,6]. Disease severity is further compounded by the organism’s propensity to form biofilms on medical equipment as well as within host tissues, contributing to persistence and therapeutic challenges [7]. Although *S. maltophilia* infections have been increasingly described across diverse patient populations, available clinical evidence remains largely derived from studies conducted in adults, with comparatively limited data describing the epidemiology, treatment patterns, and clinical outcomes of infections in pediatric patients [3,4,5,8,9,10].

Distinguishing true *S. maltophilia* infection from colonization remains a significant clinical challenge, particularly in children with chronic respiratory disease or prolonged hospitalization [11]. Although *S. maltophilia* is traditionally considered a pathogen of relatively low virulence, it is frequently isolated in the context of polymicrobial infections, further complicating efforts to determine the extent to which it contributes to a patient’s clinical presentation and need for targeted antibiotic therapy [11,12,13]. These diagnostic uncertainties underscore the importance of better characterizing the clinical significance of *S. maltophilia* isolation and treatment practices in pediatric populations [11,12,14].

Importantly, *S. maltophilia* exhibits intrinsic resistance to multiple antibiotic classes, most notably nearly all conventional beta-lactams currently available for commercial use. Its resistance to beta-lactam agents is largely mediated by the L1 and L2 beta-lactamases [11,14,15,16,17]. The L1 metallo-beta-lactamase confers resistance to penicillins, cephalosporins, and carbapenems (aztreonam remains stable against hydrolysis), whereas the L2 serine beta-lactamase activity can hydrolyze extended-spectrum cephalosporins and aztreonam. Furthermore, historically utilized first-line drugs such as trimethoprim/sulfamethoxazole (TMP/SMX), levofloxacin, and minocycline can exhibit reduced activity through upregulation of efflux pumps, and the in vitro activity of fluoroquinolones can be influenced in the setting of chromosomal Sm*qnr* genes [11,14,15,16,17].

The Infectious Diseases Society of America (IDSA) 2024 Guidance on the Treatment of Antimicrobial-Resistant Gram-Negative Infections recommendations for infections caused by *S. maltophilia* suggest combination therapy with either TMP/SMX, levofloxacin, minocycline, or cefiderocol, or ceftazidime/avibactam plus aztreonam [14]. However, evidence directly comparing monotherapy and combination therapy remains limited, and most available data are derived from adult populations [14,18]. Consequently, therapeutic decision-making in children frequently relies on extrapolation from adult studies despite important differences in drug safety, pharmacokinetics, and host susceptibility across pediatric age groups [8,9,10,14,19,20,21]. Given the limited pediatric-specific evidence describing the management of *S. maltophilia* infections, additional real-world data are needed to better characterize treatment practices and clinical outcomes in children. Therefore, the objective of this study was to describe contemporary antimicrobial treatment patterns and associated clinical outcomes among pediatric patients with *S. maltophilia* infections at a large academic medical center.

## 2. Materials and Methods

This was a retrospective, observational cohort study describing hospitalized pediatric patients at a freestanding acute care children’s hospital. Patients aged <18 years were included for review if they had any positive culture for *S. maltophilia* between January 2015 and June 2024 and received active antimicrobial therapy for confirmed or suspected infection. Patients were excluded if the organism was determined to represent colonization, as documented by the treating provider.

The primary outcome was in-hospital mortality, defined as death occurring during the same hospitalization following the index positive culture. Secondary outcomes included adverse drug effects and clinical success, defined as resolution or improvement of signs and symptoms of acute infection without modification of therapy due to clinical failure. Clinical improvement was evaluated based on objective improvements in infection-related signs and symptoms, as well as clinician-documented assessments. Descriptive statistics were used to summarize baseline characteristics and clinical outcomes. Categorical variables were reported as frequencies and percentages, and continuous variables as medians and interquartile ranges (IQRs). Statistical analyses were performed using Microsoft Excel (version 2512; Microsoft Corporation; Redmond, WA, USA). The Institutional Review Board at the Medical University of South Carolina determined this study to be exempt prior to commencement.

## 3. Results

Forty patients were included in the analysis, with a median [IQR] age of 2.7 [0.7–7.9] years. Twenty-one (52.5%) patients were female, and most patients (57.5%) were white. Common comorbidities and/or risk factors included neurological or developmental delay (57.5%), beta-lactam exposure within the preceding 90 days (95.0%), central line dependence (50.0%), and/or underlying pulmonary disease (47.5%). Most patients (67.5%) required admission to the pediatric intensive care unit (PICU) during their hospitalization, and more than half (55.0%) received an infectious diseases consultation. Hospital length of stay was a median [IQR] of 41.0 [23.3–61.5] days. Baseline demographics can be found in Table 1.

The most common sites of *S. maltophilia* isolation were endotracheal aspirate cultures (57.5%), followed by bronchoalveolar lavage (12.5%), blood (12.5%), sputum (7.5%), wound (5.0%), and cerebrospinal fluid (5.0%) cultures. Susceptibility rates for TMP/SMX and levofloxacin were 97.5% and 84.6%, respectively. The predominant infectious diagnosis was ventilator-associated pneumonia (VAP; 47.5%), followed by non-ventilator-associated hospital-acquired pneumonia identified in an additional 12 patients (30.0%) and catheter-related bloodstream infection in 5 patients (12.5%). Twenty patients (50.0%) had a concomitant polymicrobial infection during treatment for *S. maltophilia*, most commonly with Enterobacterales (n = 10; 50.0%), *Pseudomonas aeruginosa* (n = 4; 20.0%), and/or *Staphylococcus aureus* (n = 3; 15.0%).

Twenty-eight patients (70.0%) received TMP/SMX monotherapy. The median [IQR] TMP dose was 5.0 [4.8–5.6] mg/kg every 8 h (51.4%) or every 12 h (34.3%). Four patients (10.0%) were treated with levofloxacin monotherapy, and an additional four patients (10.0%) initially received TMP/SMX but were subsequently transitioned to levofloxacin due to adverse effects or provider preference/regimen consolidation. One patient (2.5%) was treated with ceftazidime/avibactam plus aztreonam. Combination therapy with TMP/SMX and levofloxacin was utilized in three patients (7.5%). Overall, the median [IQR] duration of antimicrobial therapy was 10 [7–14] days. Clinical characteristics can be found in Table 1.

The primary outcome of in-hospital mortality occurred in five patients (12.5%). None of these deaths were judged by the investigators to be due to progression or uncontrolled *S. maltophilia* infection (Table 2). All patients exhibited clinical success, and adverse drug effects attributable to TMP/SMX were documented in three patients. Two patients developed neutropenia suspected to be secondary to TMP/SMX, and one patient receiving TMP/SMX experienced interstitial nephritis. All three patients were transitioned to levofloxacin to complete their treatment course, and no additional adverse effects were reported following therapeutic alteration.

## 4. Discussion

We describe a real-world, single-center, observational experience with the management of *S. maltophilia* infections in pediatric patients. Owing to the organism’s intrinsic and acquired resistance mechanisms, therapeutic options remain limited, complicating therapeutic decision-making [11,14,15,16,17]. Although the IDSA 2024 Guidance on the Treatment of Antimicrobial Resistant Gram-Negative Infections provides treatment suggestions for both adult and pediatric populations, the management of children is frequently extrapolated from adult data due to the scarcity of pediatric-specific evidence [14]. Our report adds to the existing literature in children, which largely consists of dated case reports and small case series focused on subpopulations [8,9,10,19,20,21].

Our findings describe the clinical outcomes associated with predominantly TMP/SMX monotherapy for the treatment of *S. maltophilia* infections in children. Five patients experienced in-hospital mortality. Importantly, review of the medical records did not identify any deaths as directly attributable to progression of *S. maltophilia* infection. Furthermore, all patients demonstrated resolution or improvement of signs and symptoms consistent with presumed clinical success, and adverse effects were limited, occurring in only three individuals receiving TMP/SMX. Despite the inherent limitations of a single-center, observational design and the challenges associated with assessing outcomes in *S. maltophilia* infections, TMP/SMX monotherapy was the most frequently utilized regimen and was associated with favorable observed outcomes and a low incidence of adverse effects. Notably, most of the patients in our cohort required intensive care unit admission and had substantial underlying comorbidities, highlighting that favorable outcomes were observed despite a critically ill and clinically complex pediatric population.

Importantly, a clearly defined “standard-of-care” regimen for *S. maltophilia* infections is lacking across both adult and pediatric populations, largely due to the absence of randomized controlled trials and reliance on heterogeneous observational data [14]. In adults, comparative effectiveness studies of monotherapy versus combination therapy and across agents (e.g., TMP/SMX, fluoroquinolones, tetracycline derivatives) have yielded inconsistent results and are limited by various methodological concerns such as selection bias, small sample sizes, and variability in infection source, host factors, and treatment strategies. Available data further suggest conflicting signals between commonly used agents, including potential differences in outcomes with TMP/SMX and fluoroquinolones, though these findings are difficult to interpret due to residual confounding and study limitations [3,9,14,18,22,23,24,25]. In contrast, pediatric data are largely restricted to small case series and reports, with treatment approaches often extrapolated from adult experience. Importantly, direct extrapolation of adult treatment data may be limited by differences in underlying comorbidities, infection epidemiology, developmental pharmacokinetics, antibiotic dosing strategies, and the relative paucity of pediatric-specific effectiveness and safety data [8,10,19,20]. Collectively, these gaps highlight the persistent uncertainty in optimal antibiotic selection for *S. maltophilia* across age groups and underscore the need for rigorously designed studies to inform evidence-based recommendations.

A growing area of concern in the management of *S. maltophilia* involves emerging evidence that TMP/SMX, long considered a cornerstone therapy, may demonstrate limited activity even against isolates that test susceptible [26,27]. Recent in vitro pharmacokinetic/pharmacodynamic models have demonstrated minimal reductions in colony-forming units (CFU) at 24 h, showing bacteriostasis at best, with only 13% of experiments achieving 1-log_10_ CFU/mL reductions, even when employing supraphysiological TMP/SMX regimens (100 mg TMP/kg/day) [26]. These findings raise important questions regarding the true capacity of TMP/SMX to achieve clinically meaningful bacterial eradication. These data are concerning but must be interpreted cautiously. The findings are derived from a small number of isolates evaluated over a brief experimental period, and *S. maltophilia* has historically posed methodological challenges for in vitro susceptibility testing and experiments [26,27]. Furthermore, several observational studies have reported clinical effectiveness with TMP/SMX, albeit with notable biases and confounding inherent to these study designs [22,23,24,28]. Accordingly, additional clinical and translational studies are needed to clarify the extent to which TMP/SMX retains pharmacodynamically meaningful activity against contemporary *S. maltophilia* isolates.

*S. maltophilia* exhibits intrinsic and acquired resistance to multiple antibiotic classes through a broad array of resistance mechanisms, substantially complicating therapeutic decision-making [11,14,15,16,17]. Treatment is further challenged by issues surrounding susceptibility testing [11,14]. First, the Clinical & Laboratory Standards Institute (CLSI) has established interpretive breakpoints or susceptibility categorizations for only a small number of agents: TMP/SMX, levofloxacin, minocycline, ticarcillin/clavulanate, chloramphenicol, cefiderocol, and ceftazidime/avibactam plus aztreonam, although ticarcillin/clavulanate is no longer commercially available in the United States and chloramphenicol is rarely used in current clinical practice [14,29]. Further, robust pharmacokinetic/pharmacodynamic and clinical outcomes data, largely derived from the adult population using traditional regimens, remain limited and conflicting, and recent updates to susceptibility categorizations further complicate the interpretation of previously published reports [14,22,23,24,25,26,27,28,30,31,32,33,34]. Moreover, despite promising in vitro activity of novel agents, such as ceftazidime/avibactam plus aztreonam, aztreonam/avibactam, and cefiderocol, data evaluating their clinical effectiveness against contemporary *S. maltophilia* isolates in both the adult and pediatric populations remain limited [35,36,37,38]. As a result, defining an optimal therapeutic regimen and reliably correlating in vitro susceptibility results with clinical outcomes remains challenging [14].

Several limitations of our report should be acknowledged. First, this analysis represents a retrospective, observational single-center cohort with inherent sources of bias and a relatively small sample size, which limits the generalizability of our findings. Second, distinguishing true *S. maltophilia* infection from colonization remains inherently challenging in clinical practice, particularly from respiratory specimens which accounted for most of our cultured cases, and half of the patients in our cohort had polymicrobial infections. Consequently, although we excluded colonization as documented by the treating provider, some cases may have been misclassified as clinically significant infections, potentially leading to an overestimation of treatment success attributable specifically to *S. maltophilia*-directed therapy. Next, clinical success rates were based on documentation within the medical record and may be subject to reporting inconsistencies or incomplete capture; however, no providers altered therapy due to presumed clinical failure. As treatment selection was clinician-driven and non-protocolized, the observed predominance of TMP/SMX likely reflects local prescribing practices. Finally, the absence of a comparator group and the reliance on descriptive statistics limit the ability to draw causal inferences regarding the effectiveness of specific antimicrobial regimens.

Despite these limitations, this report provides valuable observational data on the management of *S. maltophilia* infections in pediatric patients. Our findings reflect clinical outcomes across a diverse pediatric population treated at a large academic medical center, thereby contributing meaningful real-world evidence to an otherwise limited pediatric literature base. Although TMP/SMX was the most commonly utilized therapy and was generally associated with favorable outcomes in our cohort, prospective multicenter studies are needed to better define optimal treatment strategies.

## Figures and Tables

**Table 1:** Baseline Demographics and Clinical Characteristics.

Characteristics	Results (n = 40)
Age, years, median (IQR)	2.7 (0.7–7.9)
Female	21 (52.5)
**Race**	---
White	23 (57.5)
Black	7 (17.5)
Other	10 (25.0)
**Type of ICU Admission**	---
PICU	27 (67.5)
PCICU	8 (20.0)
Hospital length of stay, days, median (IQR)	41.0 (23.3–61.5)
**Insurance Coverage**	---
Medicaid	24 (60.0)
Private	2 (5.0)
Mixed	13 (32.5)
Uninsured	1 (2.5)
**Comorbidities and Risk Factors**	---
Prematurity	11 (27.5)
Pulmonary disease	19 (47.5)
Renal disease	4 (10.0)
Neurological/developmental delay	23 (57.5)
Tracheostomy dependence	11 (27.5)
Central line dependence	20 (50.0)
Invasive procedure within last 12 months	28 (70.0)
Beta-lactam exposure within 90 days	38 (95.0)
**Infectious Diseases Consultation**	22 (55.0)
**Infection Types**	---
Ventilator-associated pneumonia	19 (47.5)
Hospital-acquired pneumonia	12 (30.0)
Catheter-related bloodstream infection	5 (12.5)
Other	4 (10.0)
Nosocomial infection	33 (82.5)
**Antimicrobial Choices**	---
TMP/SMX monotherapy	28 (70.0)
Levofloxacin monotherapy	4 (10.0)
Combination therapy	3 (7.5)

Data reported as n (%) unless noted otherwise. IQR: interquartile range; ICU: intensive care unit; PICU: pediatric intensive care unit; PCICU: pediatric cardiac intensive care unit.

**Table 2:** Clinical Characteristics and Treatment Details of Patients with In-Hospital Mortality.

Patient	Age	Notable Comorbidities	Infection Type	Treatment Regimen	Duration of Therapy
1	1.7	History of prematurity, central venous catheter dependence, end-stage renal disease secondary to dysplastic kidneys requiring peritoneal dialysis, hepatoblastoma undergoing chemotherapy, liver transplantation	Ventilator-associated pneumonia	TMP/SMX 6 mg/kg q12h	3
2	2.1	Pulmonary hypertension, central venous catheter dependence, Trisomy 21, spina bifida, scoliosis, gastrostomy dependence	Ventilator-associated pneumonia	TMP/SMX 6 mg/kg q8h	12
3	0.5	History of prematurity, central venous catheter dependence, dysplastic kidney s/p nephrostomy tube placement, uretopelvic junction obstruction	Ventilator-associated pneumonia	TMP/SMX 5 mg/kg q8h	6
4	2.5	Previously healthy, central venous catheter dependence, ARDS 2/2 recent influenza and *Streptococcus pneumoniae* infection, need for continuous renal replacement therapy and extracorporeal membrane oxygenation	Ventilator-associated pneumonia	TMP/SMX 5 mg/kg q8h	5
5	0.7	History of prematurity, central venous catheter dependence, pulmonary hypertension, ventricular septal defect, Trisomy 18, patent ductus arteriosus	Ventilator-associated pneumonia	TMP/SMX 6 mg/kg q12h	10

TMP/SMX: trimethoprim/sulfamethoxazole; ARDS: acute respiratory distress syndrome; q12h: every 12 h; q8h: every 8 h; s/p: status–post; 2/2: secondary to.

## Data Availability

The original contributions presented in this study are included in the article. Further inquiries can be directed to the corresponding author.
